# Small Molecular Prodrug Amphiphile Self-Assembled AIE Dots for Cancer Theranostics

**DOI:** 10.3389/fbioe.2020.00903

**Published:** 2020-10-02

**Authors:** Xing Yang, Yuan Luo, Sanpeng Li, Xiuli Xu, Yingxia Bao, Jiaming Yang, Defang Ouyang, Xingxing Fan, Ping Gong, Lintao Cai

**Affiliations:** ^1^Guangdong Key Laboratory of Nanomedicine, CAS-HK Joint Lab for Biomaterials, Shenzhen Institutes of Advanced Technology, Chinese Academy of Sciences, Shenzhen, China; ^2^University of Chinese Academy of Sciences, Beijing, China; ^3^Nano Science and Technology Institute, University of Science and Technology of China, Hefei, China; ^4^Guangzhou Baiyunshan Pharmaceutical Co., Ltd., Baiyunshan Pharmaceutical General Factory, Guangzhou, China; ^5^Livzon Mabpharm Inc., Zhuhai, China; ^6^State Key Laboratory of Quality Research in Chinese Medicine, Institute of Chinese Medical Sciences (ICMS), University of Macau, Macau, China; ^7^State Key Laboratory of Quality Research in Chinese Medicine, Macau Institute for Applied Research in Medicine and Health, Macau University of Science and Technology, Macau, China

**Keywords:** prodrug, self-assembly, AIE, cancer, theranostics

## Abstract

A simple and facile one-step method was developed to construct a small molecular prodrug amphiphile self-assembled organic dots CPPG with aggregation-induced emission (AIE) characteristics. Diphenylalanine peptide (FF), which is the essential moiety of the self-assembling peptide-drug conjugate and as its core recognition motifs for molecular self-assembly. In addition, the D-glucose transported protein (GLUT), which is one of the important nutrient transporters and is overexpressed in cancer cells. The conjugation of glycosyl further endues the nanoparticle with good biocompatibility and tumor-targeting ability. Taking advantages of both the cancer cell-targeting capability of small molecular prodrug amphiphile CPPG and the AIE aggregates with strong emission, the prepared CPPG AIE dots can target cancer cells specifically and inhibit the proliferation of cancer cells with good biocompatibility and photostability. Based on the general approach, types of universal organic fluorescent nanoprobes could be facilely constructed for imaging applications and biological therapeutics, which possess the properties of specific recognition and high brightness.

## Introduction

Fluorescence imaging is a low-cost and highly sensitive method for the diagnosis of early tumors, visualization of tumor margins, and evaluation of treatment effects (Ozawa et al., 2013; Weissleder and Nahrendorf, 2015; Antaris et al., 2016). A variety of nanomaterials have been developed as fluorescence nanoprobes for cancer diagnosis which bring excellent optical characteristics, as the rapid development of nanotechnology in the past few decades (Chinen et al., 2015; Wolfbeis, 2015; Fu et al., 2017). Compared with the traditional organic molecules, nanoparticles usually possess better photostability, higher brightness, and larger absorption coefficients, which make them able to enhance the versatility and sensitivity of fluorescence-based imaging and diagnosis. Unfortunately, most fluorescent nanoparticles are made by highly toxic heavy metal cations, which increase the concerns of long-time toxicity and limits further clinical transition (Tsoi et al., 2013). Considering the biocompatibility, organic fluorescent nanoparticles, composed of organic molecules decorated on a matrix of biocompatible polymers or encapsulated inside, are usually considered more suitable than the inorganic nanoparticles (Li and Liu, 2014; Yu et al., 2017).

To develop organic dye nanoparticles with significantly enhanced biocompatibility and photostability have taken great efforts (Wang et al., 2013). However, the brightness of the organic nanoparticles, gradually decrease along with the increase of dye concentration. This phenomenon has been known as aggregation-caused quenching (ACQ), which presents a barrier for fabricating organic nanoparticles (Reisch and Klymchenko, 2016). In 2001, a breakthrough in luminescent materials was made. Tang et al. reported a kind of propeller-shaped molecule with aggregation-induced emission (AIE) characteristics, such as hexaphenylsilole and tetraphenylethylene, in which the AIE luminogens generally exhibited weak or no emissions in solution but strong emissions in the solid or aggregated states (Luo et al., 2001). From then on, several AIEgens have been developed (Ding et al., 2013; Mei et al., 2015; Zhang W. et al., 2015; Yu et al., 2016; Gu et al., 2017). Furthermore, the strong emissions of the solid-state AIEgens offer a great opportunity for developing highly bright organic nanoparticles, also known as AIE dots, without blemishing their emissions (Zhang X. et al., 2015; Xu et al., 2016; Yan et al., 2016; Li H. et al., 2017; Niu et al., 2019).

A universal way of generating AIE-nanoparticles is the reprecipitation method, in which AIE molecules in gentle solvents (such as DMSO and THF) were mixed with a poor solvent (e.g., water). However, in this method, the gentle solvent is usually poisonous, which limits the applications of AIE-nanoparticles in biological environments. To solve the biocompatibility and water solubility of AIE fluorescent organic nanoparticles, the conventional methods were facilely prepared by mixing AIE material with different surfactants. A variety of strategies, such as polymerization of other monomers with AIE dyes, covalent conjugation of hydrophilic molecules with AIE dyes, and encapsulation in silica nanoparticles were considered (Zhang X. et al., 2015). The general surface functional groups facilitated further functionalization to achieve the goal of multimodal imaging, which consisted of –NH_2_, –COOH, –maleimide (–Mal) and the DSPE-PEG derivatives (distearoyl-sn-glycero-3-phosphoethanolamine poly ethylene glycol) (Li K. et al., 2013). However, the surfactant for AIE dye based nanoprobes reported previously are focused on solving the biocompatibility problem. As for the theranostic systems, use of the small molecular prodrug amphiphile, which can be self-assembled, as the surfactant of the AIE nanoprobes has not been reported yet.

**SCHEME 1:**
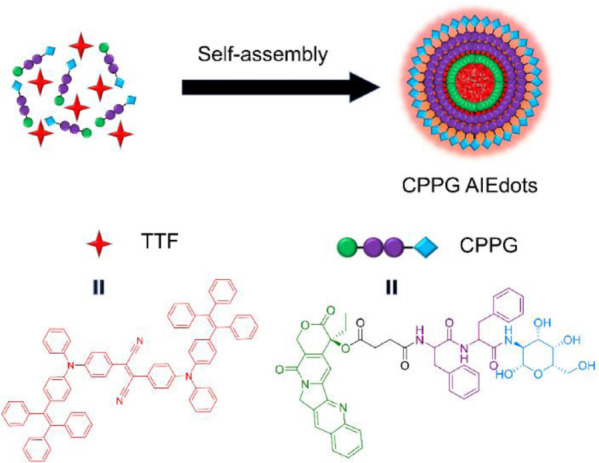
Schematic illustration of CPPG AIE dots preparation by one-pot self-assembly method.

**SCHEME 2:**
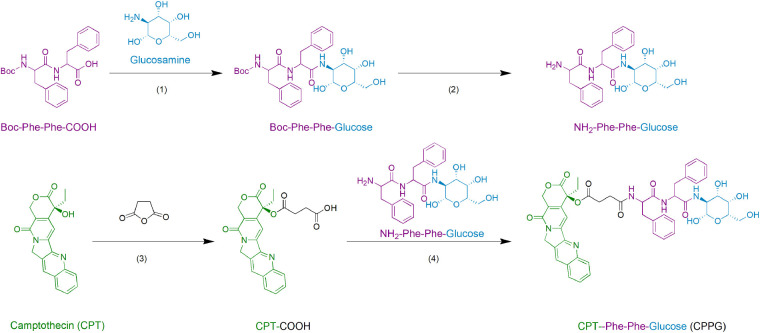
The synthesis route of small molecular drug amphiphile CPPG.

In this research, we try to develop a simple and facile way to fabricate small molecular prodrug amphiphilic AIE-dots based on a self-assembly approach which is also known as CPPG AIE dots. As illustrated in Scheme 1, the CPPG AIE dots were prepared by a one-step self-assembly method through mixing the AIEgens-TTF and CPPG under sonication. The aggregates of hydrophobic AIEgens tend to embed themselves in the hydrophobic prodrugs while the hydrophilic glucosamine chains stretch themselves into the aqueous solution to offer the AIE dots enhanced stability and good water dispersibility. The specific synthesis steps are as shown in Scheme 2.

Herein, we successfully generated the CPPG AIE dots system, the small molecular prodrug amphiphilic CPPG consists of three parts, CPT, glucosamine, and the connecting element diphenylalanine peptide (also termed as FF), and their structures were characterized by High Resolution Mass Spectrometry (HRMS), ^1^H NMR and ^13^C NMR, respectively (Supplementary Figures S1–S11). TTF, a typical AIE luminogen were added into the hydrophobic core of the CPPG, which came from Tang’s lab (Geng et al., 2014; Wang et al., 2014; Li D. et al., 2017). Camptothecin (CPT), an inhibitor of DNA topoisomerase I, (Sen et al., 2004) has been widely used to induce apoptosis under experimental conditions. Owing to its hydrophobicity, the CPT molecule acted as a self-assembly inducer by adding diphenylalanine peptide (FF), which is the essential moiety of the self-assembling peptide-drug conjugate and as its core recognition motifs for molecular self-assembly, have attracted much attention due to their biocompatibility, easy chemical modification, simple structure, and especially great capability of assembly under different conditions (Reches and Gazit, 2003; Li J. et al., 2013; Zhang H. et al., 2015). It has been widely utilized as a remarkable component to self-assemble various functional nanomaterials for biomedical applications, such as drug delivery. However, the delivery system, combining the FF with both prodrug and AIE, was rarely reported before. In addition, the D-glucose transported protein (GLUT), which is one of the important nutrient transporters and is overexpressed in cancer cells (Zhang et al., 2019). The conjugation of glycosyl further endues the nanoparticle with good biocompatibility and tumor-targeting ability (Zhao et al., 2017).

## Experimental

### Materials and Instruments

Reagents were purchased from commercial sources and were used as received unless mentioned otherwise. Reactions were monitored by TLC. ^1^H NMR and ^13^C NMR spectra were obtained by a Bruker ARX 400 MHz spectrometer in DMSO-*d*_6_. ^1^H NMR chemical shifts are reported in ppm relative to tetramethylsilane (TMS) with the solvent resonance employed as the internal standard (DMSO-*d*_6_ at 2.50 ppm). Data are reported as follows: chemical shift, multiplicity (s = singlet, br s = broad singlet, d = doublet, t = triplet, q = quartet, m = multiplet), coupling constants (Hz), and integration. ^13^C NMR chemical shifts are reported in ppm from tetramethylsilane (TMS) with the solvent resonance as the internal standard (DMSO-*d*_6_ at 39.51 ppm). High Resolution Mass Spectra (HRMS) were obtained by a GCT Premier CAB 048 mass spectrometer operating in MALDI-TOF mode. Absorption spectra were measured on a Varian 50 Conc UV-Visible spectrophotometer at 25°C. Fluorescence spectra were recorded on an Edinburgh FS5 fluorescence spectrophotometer at 25°C. Cellular imaging experiments were performed with a confocal laser scanning microscope (LSM880, ZEISS, Germany) equipped with Argon, red HeNe, and green HeNe lasers. A Cell Counting Kit-8 (CCK-8) was obtained from Dojindo Laboratories (Japan). Penicillin–streptomycin, fetal bovine serum, PBS, DMEM medium, and trypsin were acquired from Gibco Life Technologies (United States). MCF-7 and LO2 cell lines were obtained from the Shanghai cell bank of the Chinese Academy of Sciences.

### Synthesis of Compound CPT-COOH

In an ordinary vial equipped with a magnetic stirring bar, 1,8-Diazabicycloundec-7-ene (DBU, 1 mL, 0.6 mmol) was slowly at 0°C added to a mixture of (S)-(+)-camptothecin (70 mg, 0.2 mmol) and succinic anhydride (60 mg, 0.6 mmol) in 6 mL of dichloromethane. The reaction mixture was stirred at room temperature for few hours, until the reaction completed (monitored by TLC). After evaporation of the solvent, the crude product was recrystallized with methanol to obtain the pale yellow crystalline product.

^1^H NMR (400 MHz, DMSO-*d*_6_) δ 12.29 (s, 1H), 8.70 (s, 1H), 8.22 – 8.10 (m, 2H), 7.92 – 7.83 (m, 1H), 7.77 – 7.68 (m, 1H), 7.14 (s, 1H), 5.57 – 5.42 (m, 2H), 5.37 – 5.23 (m, 2H), 2.86 – 2.66 (m, 2H), 2.50 – 2.44 (m, 2H), 2.23 – 2.09 (m, 2H), 0.92 (t, *J* = 7.0 Hz, 3H).

^13^C NMR (101 MHz, DMSO-*d*_6_) δ 173.5, 171.7, 167.7, 157.0, 152.9, 148.3, 146.4, 145.7, 132.0, 130.9, 130.3, 129.5, 129.0, 128.4, 128.2, 119.4, 95.6, 76.3, 66.8, 50.7, 30.8, 29.0, 28.8, 8.0.

HRMS (ESI) calcd. for C24H20N2NaO7 [M + Na]^+^ 471.1168, found: 471.1161.

### Synthesis of Compound CPPG

Boc-Phe-Phe-Glucose: Boc-Diphenylalanine (82.4 mg, 0.2 mmol) and HATU (80.0 mg, 0.2 mmol) were dissolved in DCM (1.2 mL). Diisopropylethylamine (44 μL, 0.24 mmol) was added at 0°C, and the mixture was stirred at room temperature for 10 min. Then the solution was added to a suspension of glucosamine hydrochloride (64.7 mg, 0.3 mmol) in a mixture of DCM (1.2 mL) and diisopropylethylamine (80 μL, 0.44 mmol). After stirring at room temperature overnight, the resulting mixture was then poured into saturated brine. The insoluble solids in the dichloromethane phase were collected by centrifugation. The precipitate was washed with water (2 × 1 mL) and DCM (2 × 1 mL), and dried under vacuum to afford Boc-Phe-Phe-Glucose as a white solid.

^1^H NMR (400 MHz, DMSO-*d*_6_) δ 8.23 – 8.06 (m, 1H), 7.95 – 7.77 (m, 1H), 7.32 – 7.16 (m, 10H), 7.02 – 6.90 (m, 1H), 6.62 – 6.43 (m, 1H), 5.16 – 5.04 (m, 1H), 5.04 – 4.80 (m, 2H), 4.75 – 4.63 (m, 1H), 4.52 (s, 1H), 4.13 – 3.99 (m, 1H), 3.60 (s, 3H), 3.54 – 3.47 (m, 1H), 3.18 – 3.07 (m, 2H), 2.88 – 2.76 (m, 2H), 2.69 (s, 1H), 2.66 – 2.56 (m, 1H), 1.27 (s, 9H).

^13^C NMR (101 MHz, DMSO-*d*_6_) δ 171.5, 155.5, 138.7, 138.1, 130.1, 130.0, 129.6, 128.4, 128.3, 128.3, 126.5, 91.1, 78.6, 77.3, 72.6, 71.4, 71.2, 70.8, 61.5, 56.6, 55.0, 53.8, 49.0, 38.9, 38.7, 38.0, 28.5, 28.2.

HRMS (ESI) calcd. for C29H39N3NaO9 [M + Na]^+^ 596.2584, found: 596.2568.

Procedure for the Boc Deprotection: Boc-Phe-Phe-Glucose (28.7 mg, 0.05 mmol) was dissolved in DCM (1 mL) and cooled to 0°C. TFA (trifluoroacetic acid) (2 mL, 26 mol) was added dropwise, and the stirring was continued for 1 h at room temperature. After the reaction was completed, the solvent and TFA were removed by evaporation. To the residue, cold diethyl ether was added whereupon the product precipitated. The product was filtered off via a sinter funnel and dried in a vacuum leaving the Phe-Phe-Glucose trifluoroacetate as a white powder.

To compound CPT-COOH (22.4 mg, 0.05 mmol) in 1 mL anhydrous DMSO the following was added: NHS (N-Hydroxysuccinimide) (6.9 mg, 0.06 mmol), DIC (N,N′-Diisopropylcarbodiimide) (7.6 mg, 0.06 mmol), and DMAP (4-Dimethylaminopyridine) (7.3 mg, 0.06 mmol), at room temperature. After the reaction was stirred for 12 h at room temperature, Phe-Phe-Glucose trifluoroacetate (29.3 mg, 0.05 mmol) was added, and then stirred for 2 h. The resulting mixture was then poured into DCM and deionized H_2_O. The insoluble solids in the dichloromethane phase were collected by centrifugation. The precipitate was washed with water and DCM, and dried under a vacuum to create CPPG as a pale yellow solid.

^1^H NMR (400 MHz, DMSO-*d*_6_) δ 8.74 – 8.67 (m, 1H), 8.22 – 8.13 (m, 3H), 7.92 – 7.80 (m, 2H), 7.76 – 7.70 (m, 1H), 7.28 – 7.21 (m, 2H), 7.22 – 7.09 (m, 10H), 7.09 – 7.04 (m, 1H), 6.71 – 6.65 (m, 1H), 5.56 – 5.43 (m, 3H), 5.33 – 5.23 (m, 2H), 4.95 (s, 1H), 4.73 – 4.37 (m, 4H), 3.66 – 3.55 (m, 3H), 3.52 – 3.46 (m, 1H), 3.00 (s, 3H), 2.72 (s, 2H), 2.37 (s, 2H), 2.35 – 2.30 (m, 2H), 2.10 – 2.04 (m, 2H), 0.89 (t, *J* = 7.0 Hz, 3H).

^13^C NMR (101 MHz, DMSO-*d*_6_) δ 173.3, 171.7, 171.4, 170.7, 167.7, 157.0, 152.8, 148.4, 147.4, 146.4, 145.8, 138.4, 138.2, 132.0, 130.9, 130.2, 129.9, 129.6, 129.5, 129.0, 128.4, 128.4, 128.3, 126.6, 119.2, 107.2, 95.6, 91.2, 76.5, 76.2, 72.6, 71.6, 71.0, 66.8, 61.6, 54.8, 54.4, 54.0, 50.7, 47.8, 38.4, 37.7, 30.8, 30.0, 29.4, 25.7, 23.8, 8.0.

HRMS (ESI) calculated for C_48_H_49_N_5_NaO_13_ [M + Na]^+^ 926.3225, found: 926.3196.

### Preparation and Characterization of CPPG AIE Dots

The as-prepared hydrophobic AIEgens (2,3-bis(4-(phenyl(4-(1,2,2-triphenylvinyl) phenyl) amino) phenyl) fumaronitrile), also known as TTF, were transferred into an aqueous solution by coating with a CPPG amphiphilic molecular. A combination of 1 mg CPPG and 1 mg TTF were then dissolved in tetrahydrofuran (THF), then CPPG was added dropwise to water under an ultrasonic environment, and repeatedly pipetted with a pipette to remove THF, so that the amphiphilic molecules self-assembled to form a water-soluble nano skeleton. Then, under the same conditions, according to the volume of 1:1, an equal volume of the TTF solution was drawn, added drop by drop, and repeatedly pipetted to remove THF to make amphiphilic molecules through self-assembly to form a water-soluble nano skeleton. The liquid in the beaker was then transferred to a dialysis bag with a molecular weight of 1 KD, and dialyzed against distilled water for 48 h, during which the water was changed every 8 h to remove free single molecules that did not form particles. Then the purified CPPG AIE dots were concentrated for further characterization and application. The UV–vis absorbance spectra and photoluminescence (PL) spectra of CPPG AIE dots were measured using PerkinElmer Lambda 25 UV–vis absorption spectrophotometer and Edinburgh FS920 fluorescent spectrometer, respectively. TEM images of CPPG AIE dots were recorded using the FEI Tecnai G20 transmission microscope at 200 kV. Dynamic light scattering (DLS) analysis was taken using a Zetasizer Nano ZS (Malvern Instruments).

### Cell Treatment and Cell Imaging

For the imaging of AIE, the MCF7 and LO2 cells were incubated with CPPG AIE dots at 37°C. For glucosamine pre-block cell imaging, a solution of glucosamine (10 mg/mL) was prepared (dissolved in DMEM) and added to each pore, then incubated at 37°C for 30 min. The imaging was acquired using a confocal laser scanning microscope (LSM 880, ZEISS, Germany). For cell imaging, the cells were washed with PBS three times. A 480 nm laser was used as the light source and emission was collected from 600 to 700 nm.

### Cell Viability

Cell viability was determined by a CCK-8 assay. Firstly, 150 μL cell suspension was prepared in a 96-well plate and incubated in the incubator for 24 h (37°C, 5% CO_2_). Then 2 μL of different concentrations of (0–10 mg/mL) CPT and CPPG-AIE were, respectively added to the plate and incubated for 48 h. After removing the solution, 100 μL 10% CCK-8 (dissolved in DMEM) was added to each pore (attention was paid to not generate bubbles) and incubated for 40 min. Finally, the absorbance at 450 nm was measured by a microplate reader. Cell Survival Rate = [(As-Ab)/(Ac-Ab)] × 100% [As: Laboratory pore (medium containing cells, CCK-8, CPT, and CPT or CPPG-AIE); Ac: Control pore (medium containing cells, CCK-8, without CPT or CPPG-AIE); Ab: Blank pore (medium without cells and CPT or CPPG-AIE, CCK-8)].

### Statistical Analysis

All the results are reported as mean ± SD. The differences among groups were determined using one-way ANOVA analysis and student’s *t*-test.

## Results and Discussion

### Characteristics of CPPG AIE Dots

**FIGURE 1:**
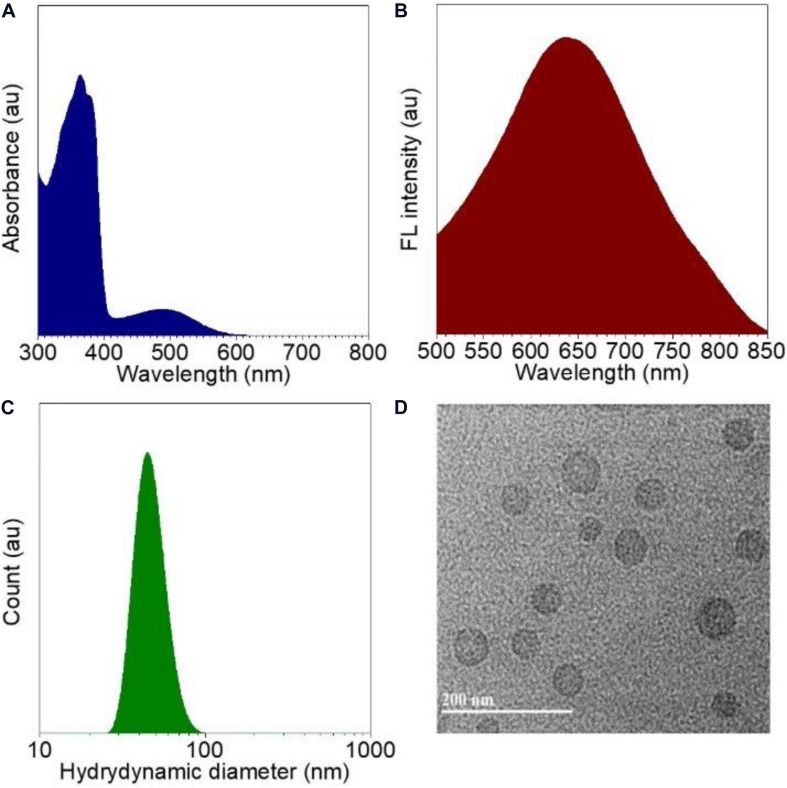
Characterizations of CPPG AIE dots. **(A)** Absorption spectra recorded for CPPG AIE dots. **(B)** Fluorescence emission spectra for CPPG AIE dots. **(C)** Hydrodynamic diameter distribution obtained for CPPG AIE dots. **(D)** TEM image obtained for CPPG AIE dots. Synthesis route of small molecular drug amphiphile CPPG.

With the CPPG AIE dots in hand, we first investigated the spectral characteristics, all the test samples are from 4 mg/mL CPPG AIE dots, the UV/Vis absorption spectrum of CPPG AIE dots dispersion displayed typical signals of TTF at ∼480 nm and the peak of CPPG at ∼370 nm (Figure 1A and Supplementary Figure S12), as for the fluorescence emission spectra, CPPG AIE dots show a emission peak at ∼650 nm (Figure 1B), which further confirmed the successfully synthesized of CPPG AIE dots. Furthermore, DLS analysis revealed that the average diameter of CPPG AIE dots was ∼57 nm (Figure 1C), the uniform spherical morphology was confirmed by transmission electron microscopy (TEM) analysis (Figure 1D). In terms of stability, the size of CPPG AIE dots stabilized within a week (Supplementary Figure S13) and the fluorescence intensity remains unchanged under excitation light which indicated good photostability (Supplementary Figure S14). To study the fluorescence performance of AIE dots in the poor solvent, we changed the fraction of water and THF (Supplementary Figure S15) which showed poor solubility when the percentage of water increased. In addition, to study the loading capacity and versatility of the nanodots formed by CPPG, we tested the different volume mixing ratio of CPPG with TTF (Supplementary Figures S16A,B) and two other AIEgens in the CPPG dots, they are DCPP-TPA (Supplementary Figures S16C,D) and MEH-PPV (Supplementary Figures S16E,F), respectively. The results indicated that the optimal loading capacity when the mixing ratio is 1:1.

### Cell Imaging of CPPG AIE Dots

**FIGURE 2:**
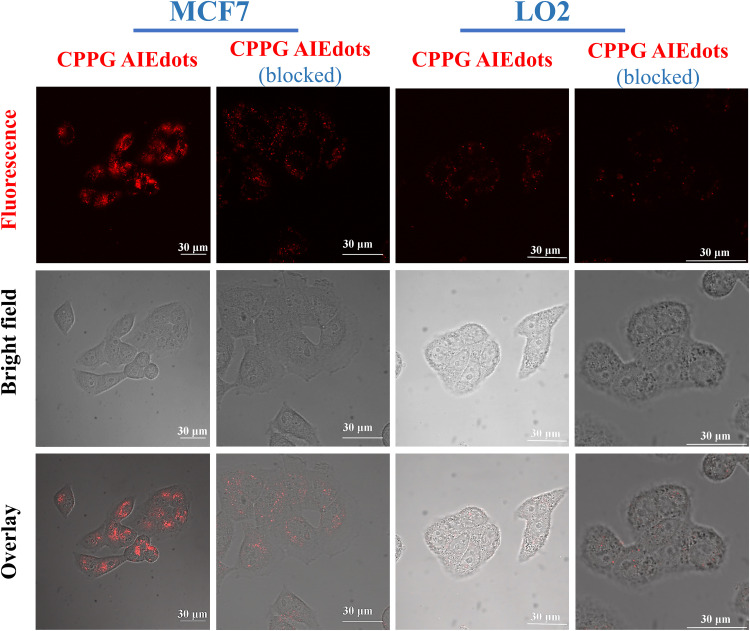
Cancer cell targeting property of CPPG AIE dots. CLSM (Confocal Laser Scanning Microscopy) images of MCF7 cells (GLUT high-concentration) and LO2 cells (GLUT low-concentration) after incubating with CPPG AIE dots under different treatments for 3 h at 37°C. For the pre-blocking experiment, cells were pretreated with 10 mg/mL free glucosamine hydrochloride (dissolved in DMEM) for 30 min before incubation with CPPG AIE dots.

**FIGURE 3:**
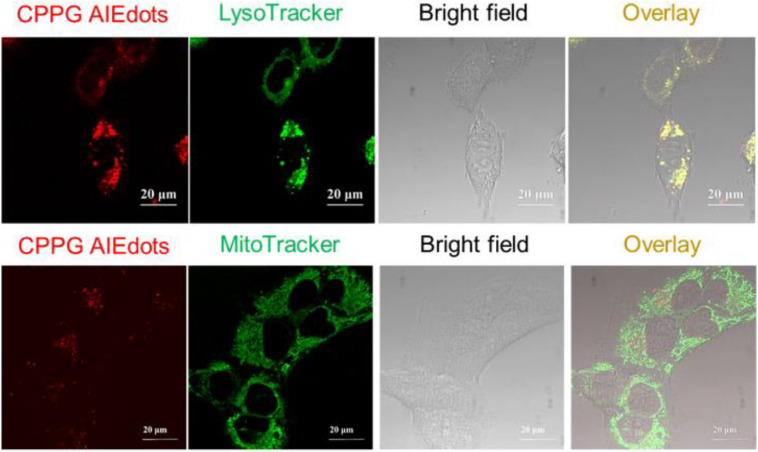
Colocalization studies of CPPG AIE dots with LysoTracker Green DND-99 or MitoTracker Green FM in MCF7 cells, respectively.

Since GLUT presents high concentration in cancer cells, the property of CPPG AIE dots as cancer cell specific fluorescence light-up probes was studied for live cell imaging. Herein, the human breast cancer cell line MCF7 and human hepatocytes normal cell line LO2 were selected as the model for fluorescence imaging research. As displayed in Figure 2, under a fluorescence microscope, a strong red fluorescence was detected after MCF7 cells were incubated with 4 mg/mL CPPG AIE dots. However, compared to the MCF7 cells, a weak red fluorescence was observed in LO2 cells. Furthermore, when both MCF7 and LO2 cells were pre-treated with excess free glucosamine hydrochloride for 30 min before the addition of CPPG AIE dots, only a very weak red fluorescence was detected (Figure 2). These results suggested that the intracellular fluorescence light-up of CPPG AIE dots almost came from the endocytosis mediated by the GLUT pathway. Moreover, the localization of CPPG AIE dots was tracked by Lysotracker and Mitotracker (Figure 3), as it matched very well with the Lysotracker, which also suggested that the entry of CPPG AIE dots into cells was at least partially GLUT receptor-mediated endocytosis. Herein, CPPG AIE dots can aggregated much more in the cancer cells, which revealed a potential way to distinguish cancer cell from normal cells.

### Cell Viability

**FIGURE 4:**
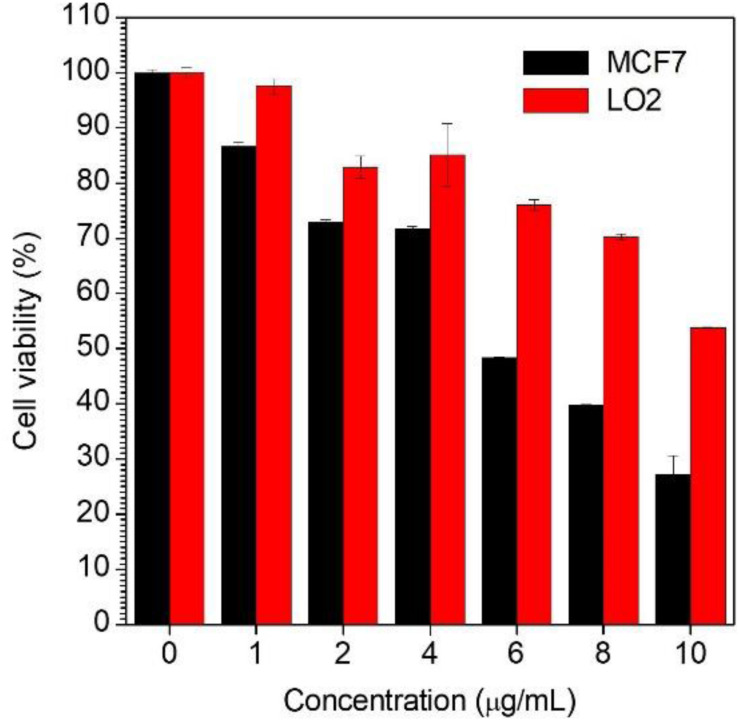
Cell viability determined by CCK-8 assay against MCF7 cells and LO2 cells upon 48 h treatment with CPPG-AIE dots.

In order to assess the possibility of using the AG-targeted prodrug CPPG AIE dots to inhibit cancer cells, the cell viabilities of MCF7 and LO2 were studied using CCK8 (Cell Count Kits-8). As is shown in Figure 4, when treated with the same concentration of the CPPG AIE dots, the cell viability of MCF7 was much lower than LO2, which suggests that our design of nanoprobe CPPG AIE dots works well in targeting the cancer cell and also significantly inhibiting the cell growth.

## Conclusion

In summary, taking advantage of the characteristics of the AIEgens, we have successfully synthesized AIE-based intracellular light-up nanoprobe CPPG AIE dots with self-assemble and cancer cell targeted prodrug properties. The CPPG AIE dots nanoprobe was selected for cancer cell targeted imaging and selective suppression of the growth of cancer cells. As far as we know, the strategies to combine the AIEgens with prodrug by the FF element have not been widely used yet. Herein, such an AIE-based nanoprobe offers a new choice for the development of fluorescence light-up imaging and anticancer therapeutics.

## Data Availability Statement

The raw data supporting the conclusions of this article will be made available by the authors, without undue reservation.

## Author Contributions

XY and YL: data curation, validation, visualization, investigation and writing–original draft. SL, XX, YB, JY, DO, and XF: writing-original draft. PG and LC: funding acquisition, supervision, project administration, methodology, and writing–review and editing.

## Conflict of Interest

YB was employed by the company Guangzhou Baiyunshan Pharmaceutical General Factory. JY was employed by the company Livzon Mabpharm Inc., Zhuhai, China. The remaining authors declare that the research was conducted in the absence of any commercial or financial relationships that could be construed as a potential conflict of interest.
