# Supplementary material for: Small Molecular Prodrug Amphiphile Self-Assembled AIE Dots for Cancer Theranostics
Source: Front Bioeng Biotechnol. 2020 Oct 2;8:903. doi: 10.3389/fbioe.2020.00903 (PMC7566912; doi:10.3389/fbioe.2020.00903)
Supplement: Supplementary file 1 [file Data_Sheet_1.pdf]

Supplementary Material

Small Molecular Prodrug Amphiphile Self-assembled AIE dots for Cancer Theranostics

Xing Yang[1,2‡], Yuan Luo[1,‡], Sanpeng Li[1,‡], Xiuli Xu[1,3], Yingxia Bao[4], Jiaming Yang[5], Defang Ouyang[6], Xingxing Fan[7], Ping Gong [1*], Lintao Cai [1*]

[1] Guangdong Key Laboratory of Nanomedicine, CAS-HK Joint Lab for Biomaterials, Shenzhen Institutes of Advanced Technology, Chinese Academy of Sciences, Shenzhen, China

[2] University of Chinese Academy of Sciences, Beijing, China

[3] Nano Science and Technology Institute, University of Science and Technology of China, Hefei, China

[4]Guangzhou Baiyunshan Pharmaceutical Co. Ltd., Baiyunshan Pharmaceutical General Factory, Guangzhou, China

[5] Livzon Mabpharm Inc., Zhuhai, China

[6] State Key Laboratory of Quality Research in Chinese Medicine, Institute of Chinese Medical Sciences (ICMS), University of Macau, Macau, China

[7] State Key Laboratory of Quality Research in Chinese Medicine, Macau Institute for Applied Research in Medicine and Health, Macau University of Science and Technology, Macau, China.

* **Correspondence:** Prof. Lintao Cai (E-mail: lt.cai@siat.ac.cn) and Prof. Ping Gong (ping.gong@siat.ac.cn)

‡ These authors contributed equally to this work.

Keywords: prodrug, self-assembly, AIE, cancer, theranostics

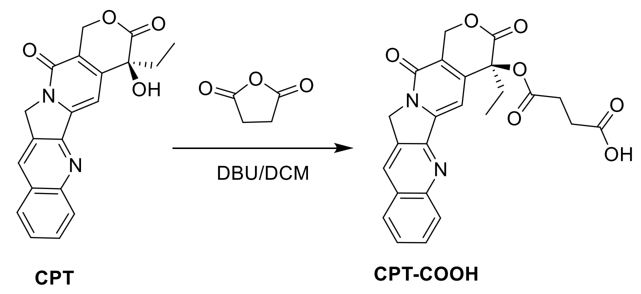

Supplementary Figure 1: Synthetic route to **CPT-COOH** .

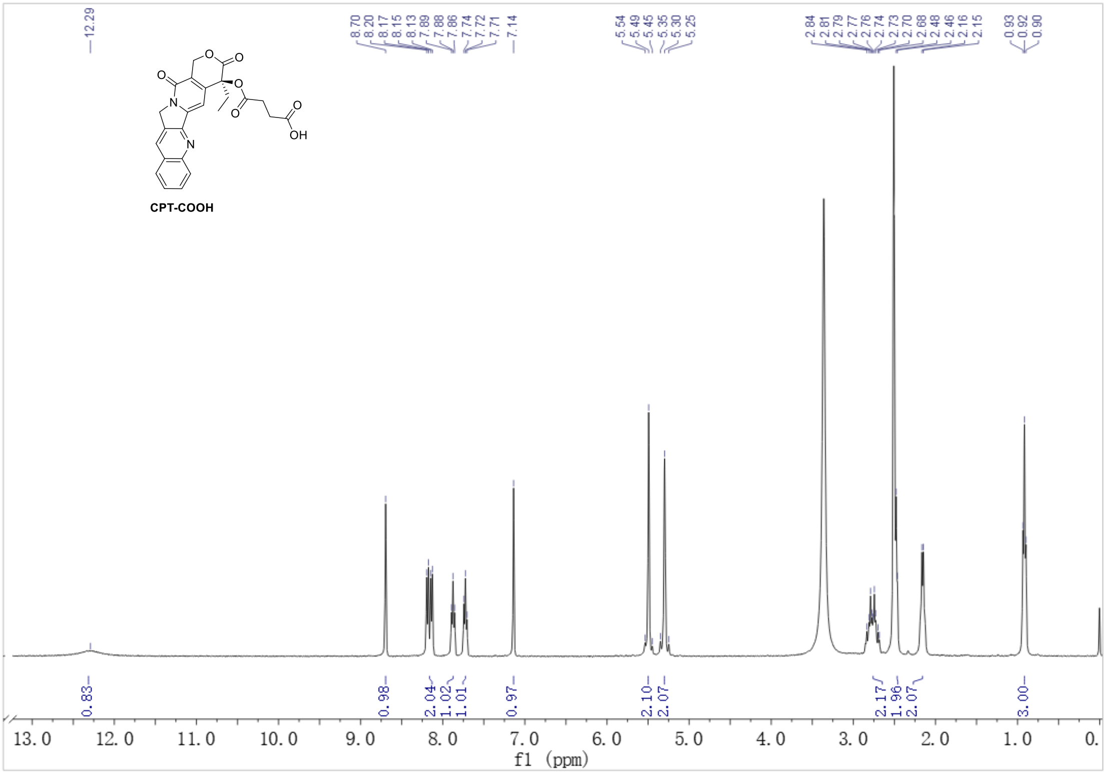

Supplementary Figure 2: ^{1}H NMR spectrum of **CPT-COOH.**

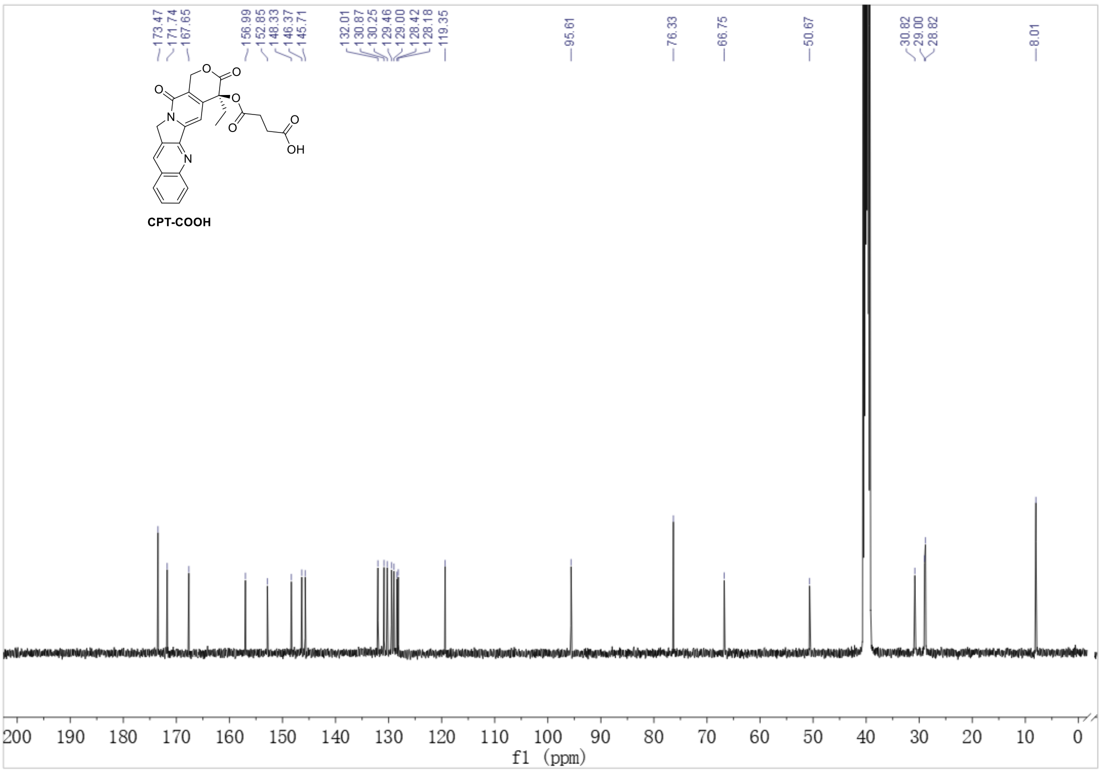

Supplementary Figure 3: ^{13}C NMR spectrum of **CPT-COOH**.

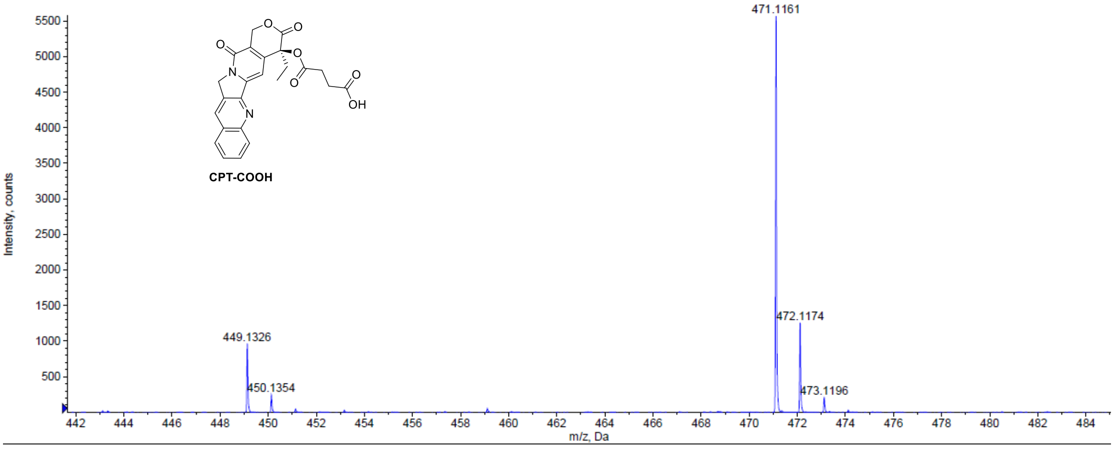

Supplementary Figure 4: High resolution mass spectrum (MALDI-TOF) of **CPT-COOH**.

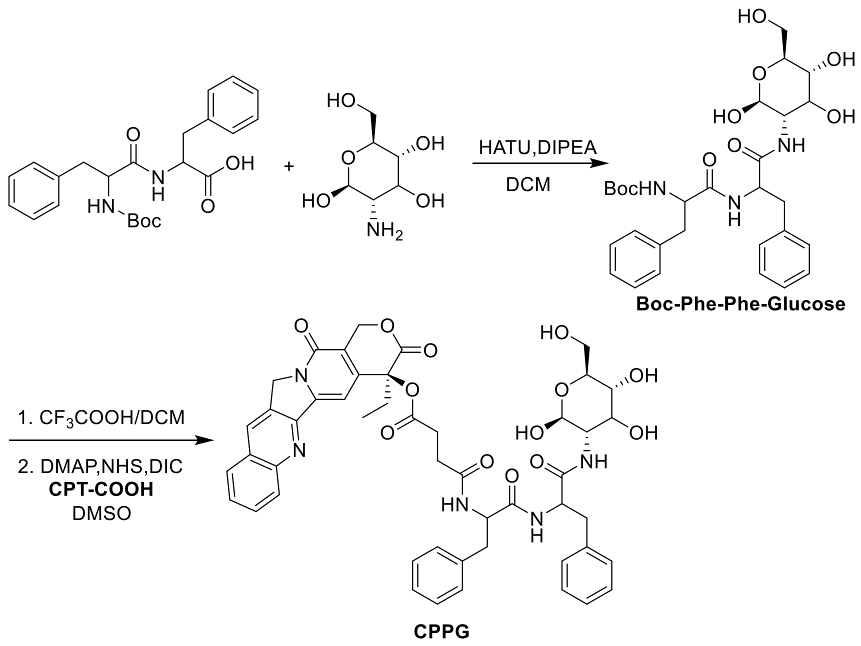

Supplementary Figure 5: Synthetic route to **Boc-Phe-Phe-Glucose** and **CPPG.**

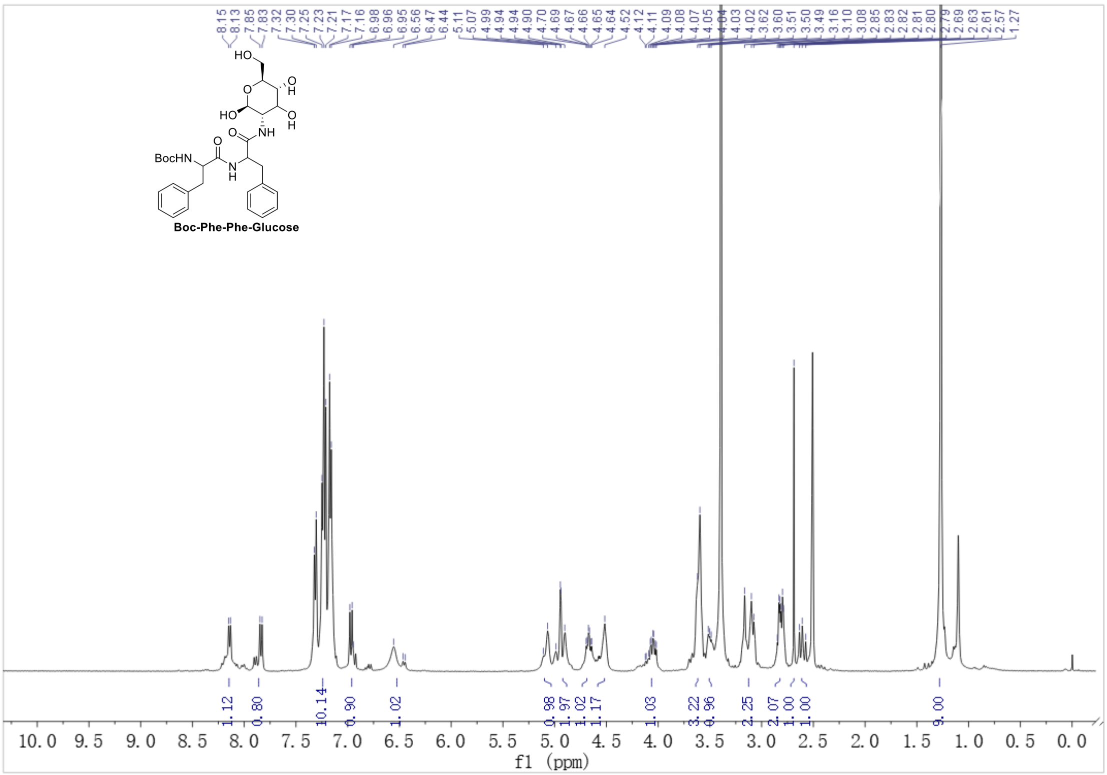

Supplementary Figure 6: ^{1}H NMR spectrum of **Boc-Phe-Phe-Glucose.**

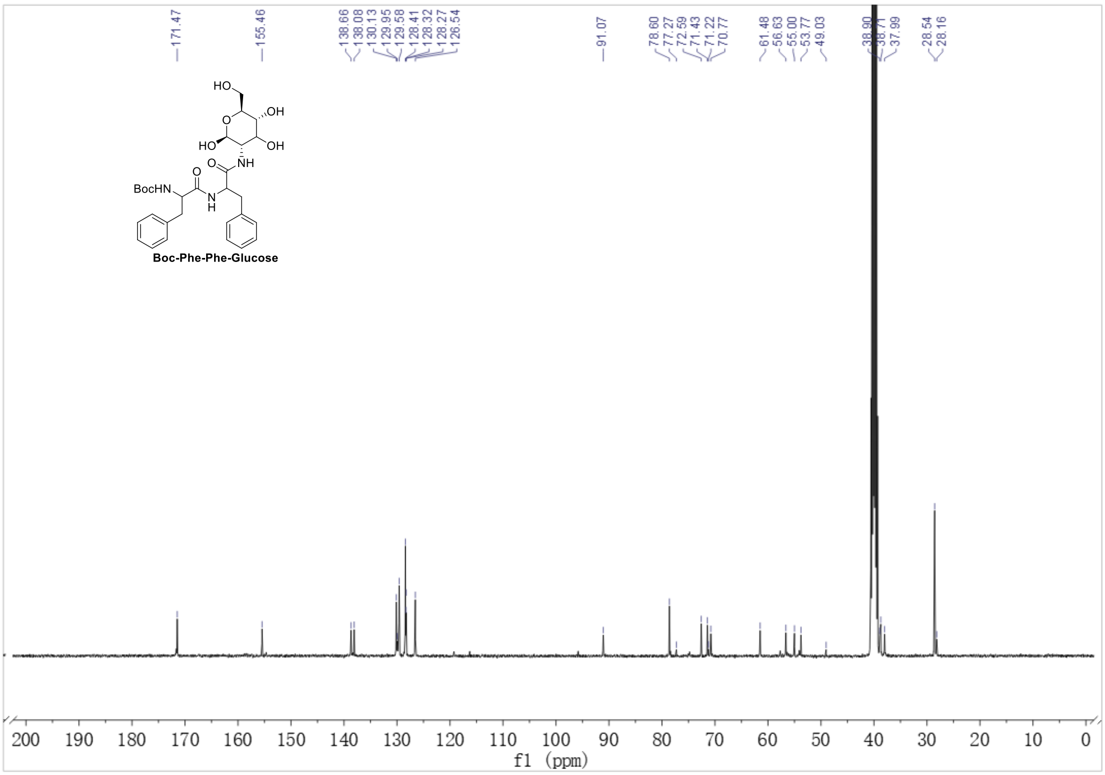

Supplementary Figure 7: ^{13}C NMR spectrum of **Boc-Phe-Phe-Glucose.**

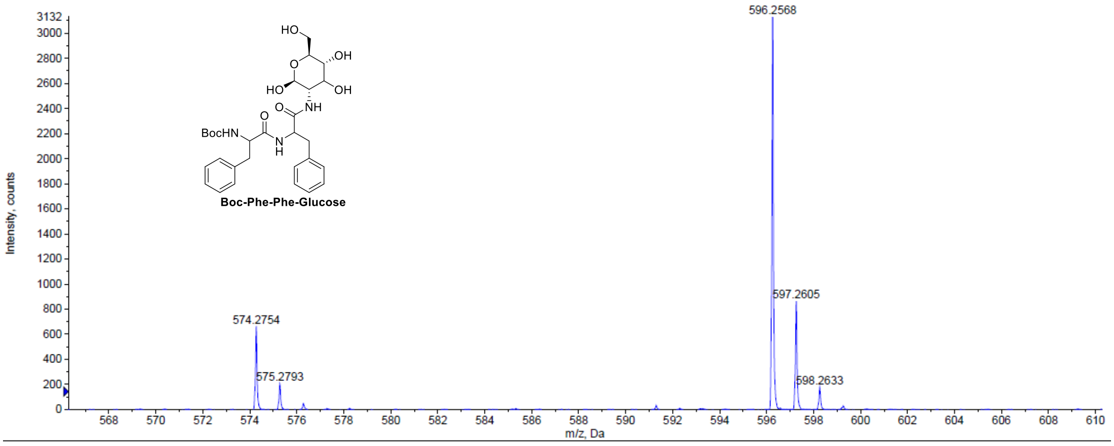

Supplementary Figure 8: High resolution mass spectrum (MALDI-TOF) of **Boc-Phe-Phe-Glucose.**

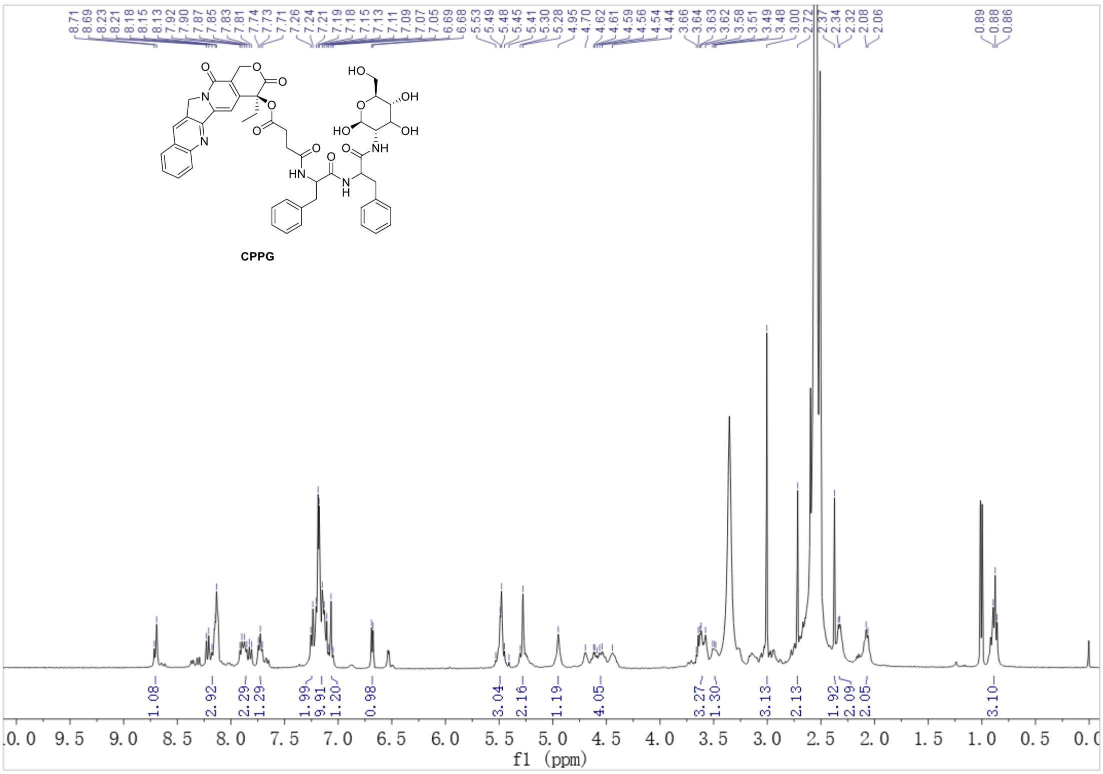

Supplementary Figure 9: ^{1}H NMR spectrum of **CPPG.**

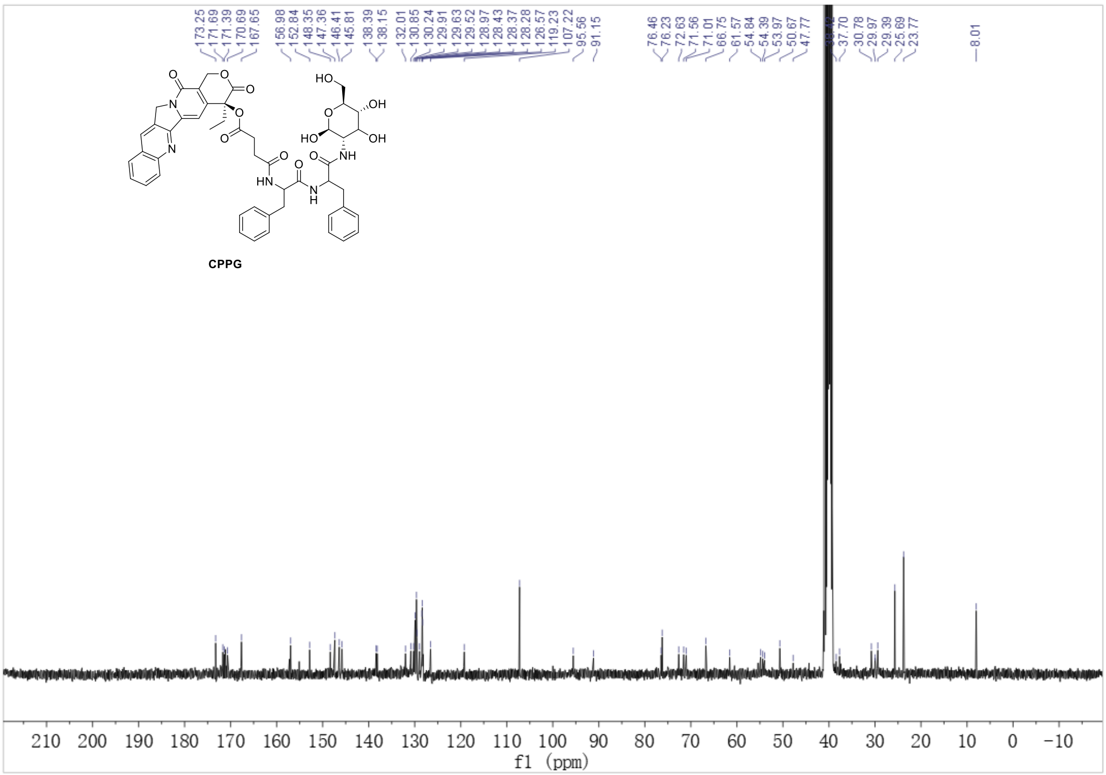

Supplementary Figure 10: ^{13}C NMR spectrum of **CPPG.**

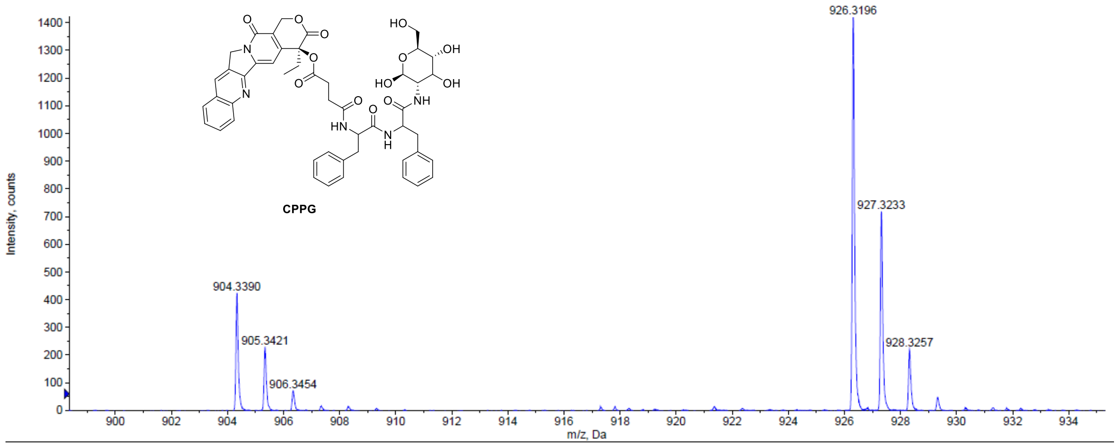

Supplementary Figure 11: High resolution mass spectrum (MALDI-TOF) of **CPPG.**

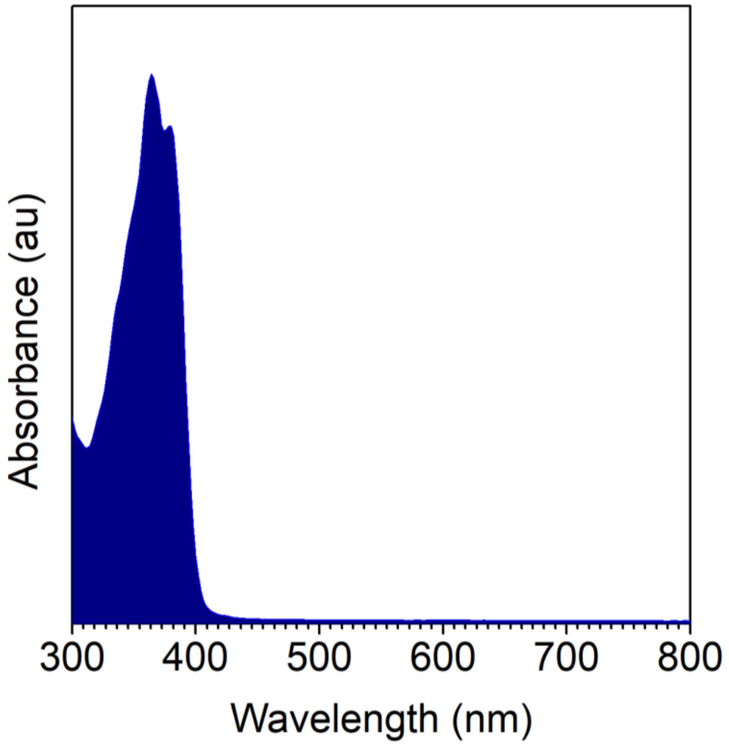

Supplementary Figure 12: Absorption spectra recorded for free **CPPG.**

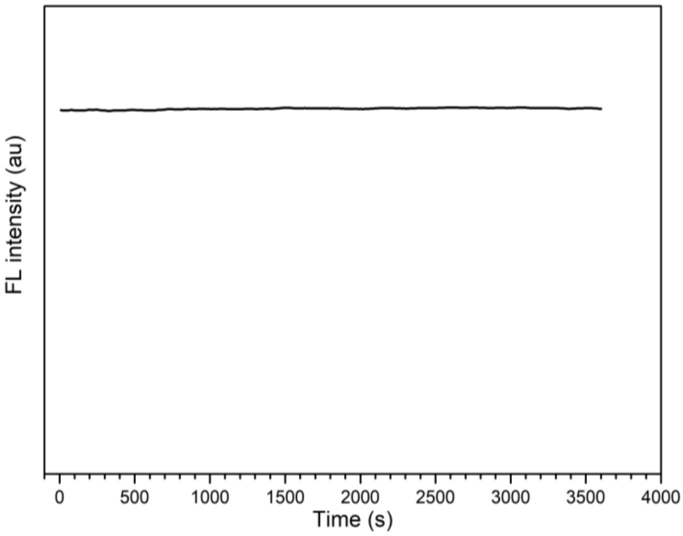

Supplementary Figure 13: Photostability of CPPG AIE.

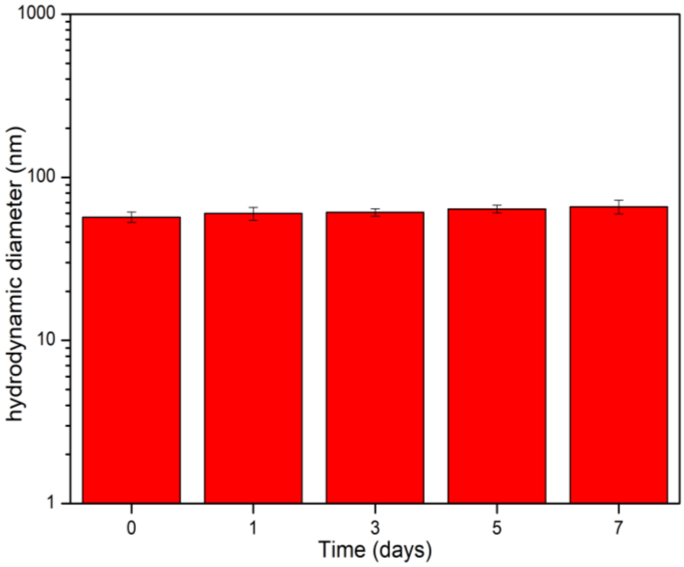

Supplementary Figure 14: Stability of CPPG AIE in DI water.

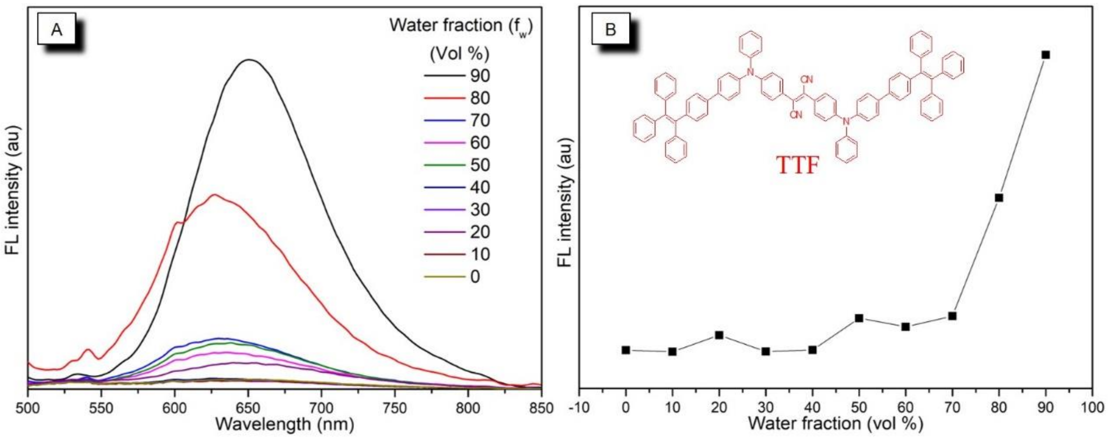

Supplementary Figure 15: The fluorescence intensity of TTF in the mixture of water and THF.

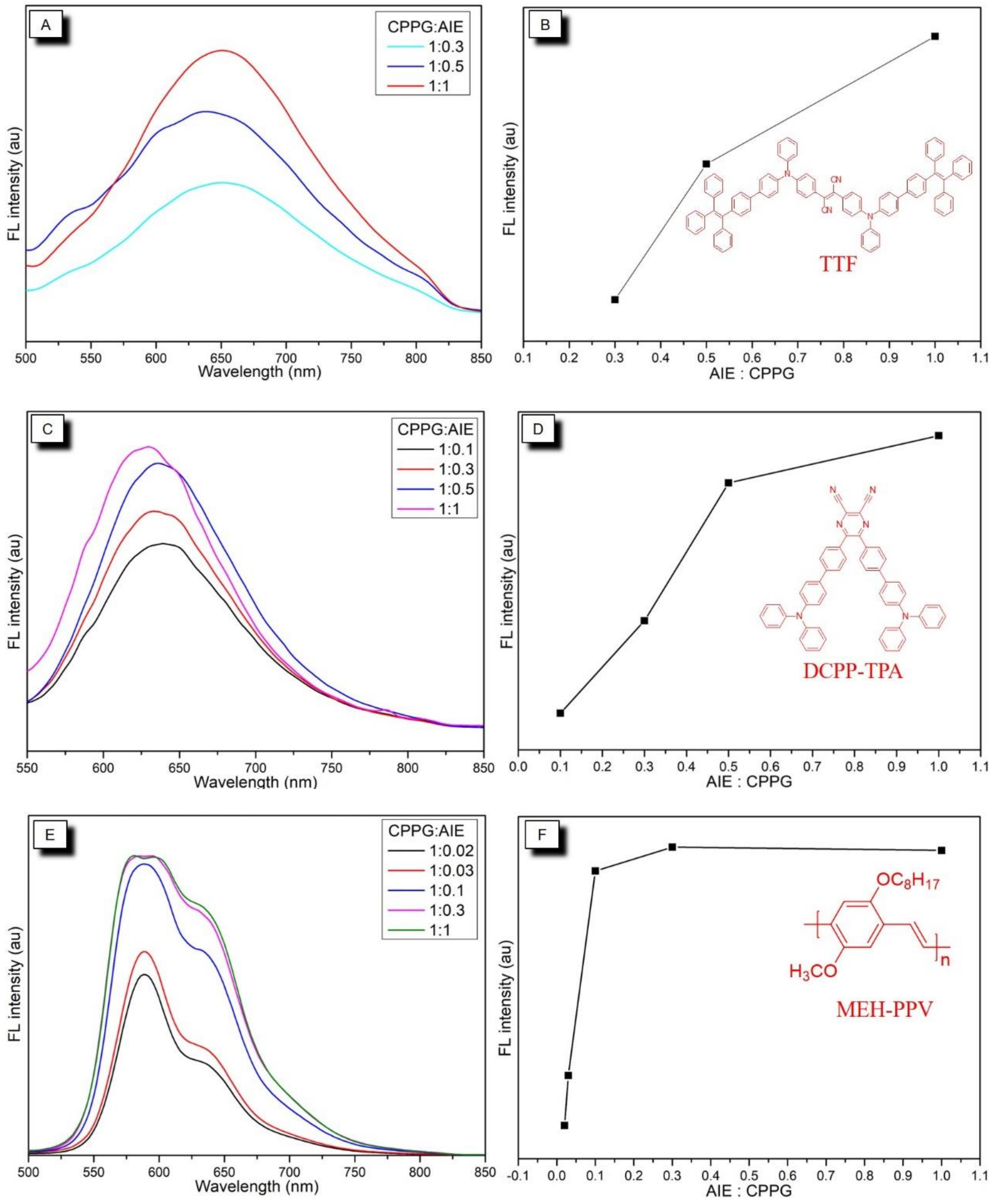

Supplementary Figure 16: The fluorescence intensity and loading capacity of different AIEgens formed by self-assembly of CPPG. **(A-B)** The fluorescence intensity and loading capacity of CPPG TTF. **(C-D)** The fluorescence intensity and loading capacity of CPPG TPA. **(E-F)** The fluorescence intensity and loading capacity of CPPG MEH-PPV.
